# Differential Responses to Low- and High-Frequency Subthalamic Nucleus Deep Brain Stimulation on Sensor-Measured Components of Bradykinesia in Parkinson’s Disease

**DOI:** 10.3390/s24134296

**Published:** 2024-07-02

**Authors:** Akash Mishra, Vikram Bajaj, Toni Fitzpatrick, Jeremy Watts, Anahita Khojandi, Ritesh A. Ramdhani

**Affiliations:** 1 Department of Neurology, Donald and Barbara Zucker School of Medicine at Hofstra/Northwell, 300 Community Drive, Manhasset, NY 11030, USA; 2 Department of Industrial and Systems Engineering, University of Tennessee, Knoxville, TN 37996, USA

**Keywords:** deep brain stimulation, subthalamic nucleus, bradykinesia, sensors

## Abstract

Introduction: The current approach to assessing bradykinesia in Parkinson’s Disease relies on the Unified Parkinson’s Disease Rating Scale (UPDRS), which is a numeric scale. Inertial sensors offer the ability to probe subcomponents of bradykinesia: motor speed, amplitude, and rhythm. Thus, we sought to investigate the differential effects of high-frequency compared to low-frequency subthalamic nucleus (STN) deep brain stimulation (DBS) on these quantified facets of bradykinesia. Methods: We recruited advanced Parkinson’s Disease subjects with a chronic bilateral subthalamic nucleus (STN) DBS implantation to a single-blind stimulation trial where each combination of medication state (OFF/ON), electrode contacts, and stimulation frequency (60 Hz/180 Hz) was assessed. The Kinesia One sensor system was used to measure upper limb bradykinesia. For each stimulation trial, subjects performed extremity motor tasks. Sensor data were recorded continuously. We identified STN DBS parameters that were associated with improved upper extremity bradykinesia symptoms using a mixed linear regression model. Results: We recruited 22 subjects (6 females) for this study. The 180 Hz STN DBS (compared to the 60 Hz STN DBS) and dopaminergic medications improved all subcomponents of upper extremity bradykinesia (motor speed, amplitude, and rhythm). For the motor rhythm subcomponent of bradykinesia, ventral contacts yielded improved symptom improvement compared to dorsal contacts. Conclusion: The differential impact of high- and low-frequency STN DBS on the symptoms of bradykinesia may advise programming for these patients but warrants further investigation. Wearable sensors represent a valuable addition to the armamentarium that furthers our ability to conduct objective, quantitative clinical assessments.

## 1. Introduction

The hallmark symptom of Parkinson’s Disease (PD) is bradykinesia, or a slowness of movement. Features of bradykinesia can be associated with other motor phenomenon, including hypokinesia (reduction in movement amplitude), sequencing effects (progressive reductions in amplitude and velocity), and irregularities in movement timing [1]. The measurement of bradykinesia relies on the Movement Disorder Society Unified Parkinson’s Disease Rating Scale (MDS-UPDRS), which places weights on these various features equally. This allows for the potential diagnosis of bradykinesia in an individual with only one of the motor features. Further, dopaminergic medication and high-frequency deep brain stimulation (DBS) produce improvement in movement velocity and amplitude without any evidence of sequencing effects or arrhythmicity responses [2,3,4]. This suggests that distinct pathophysiological mechanisms underlie aspects of bradykinesia in the motor network. 

While high-frequency deep brain stimulation of the subthalamic nucleus (STN) is commonly utilized for treating the motor symptoms of PD, low-frequency stimulation (<100 Hz) has been shown to improve limb bradykinesia as well [5,6]. Inertial sensors offer objective measures of bradykinesia by calculating the mean acceleration of movement from either a uniaxial or bi-axial accelerometer recording movement at a 1–3.5 Hz signal range [7]. This allows for the disentanglement of voluntary movements from tremor. One such sensor is the Kinesia ONE (Great Lake Neurotechnologies, Cleveland, OH, USA), which can quantify the changes in the speed, amplitude, and rhythm of distal motor impairment in PD patients. This system has been validated against the MDS-UPDRS III motor subscale for bradykinesia with high test reliability and sensitivity [8] and demonstrated to be more precise in evaluating the differences in motor amplitude and speed [9]. Recent studies utilized simple repetitive movements such as finger tapping to identify the relationship between sensor metrics and UPDRS scores [10] and there is an ongoing effort to automate UPDRS calculations from such movements [11]. 

Hence, we conducted a study with the following aims: (1) determine if low-frequency (60 Hz) STN DBS differentially impacts limb bradykinesia compared to high-frequency (180 Hz) STN DBS using the Kinesia inertial sensor and (2) decipher whether the differential effects of stimulation frequency on bradykinesia are impacted by stimulation location or medication state. 

## 2. Methods

### 2.1. Study

This study was conducted as part of a larger clinical trial that investigated the effects of 60 Hz and 180 Hz STN DBS on gait disorder in advanced PD patients. The trial was approved by the institutional review boards of The Feinstein Institutes for Medical Research (Northwell Health) and the University of Tennessee at Knoxville and registered as a clinical trial (NCT #04184791). This study’s methodology, as it pertains to gait outcomes, was previously reported [12].

### 2.2. Subjects

All participants provided written informed consent to participate in this study. We recruited patients who had undergone the implantation of four-ring contact DBS leads in their bilateral STN at least three months prior to study enrollment for the treatment of advanced idiopathic PD. All patients were diagnosed with PD according to clinical criteria. Subjects were excluded from the study if they exhibited cognitive deficits that limited their compliance with the study protocol. The implanted DBS systems were St. Jude Medical Infinity (Abbott Laboratories, Chicago, IL, USA) or Medtronic (Medtronic Inc., Dublin, Ireland). 

### 2.3. Procedures

We implemented a single-blind stimulation trial. The Kinesia sensor system (a motion sensor consisting of a three-dimensional gyroscope and accelerometer) was placed on subjects’ fingers and ankles (bilateral). While seated, subjects were guided through a series of movement tasks by interacting with the Kinesia system tablet. The instructions for each motor task appeared on the tablet screen. Subjects performed two tasks to assess upper limb bradykinesia: a hand grasp task and an arm pronation–supination movement. During these tasks, the sensor system continuously captured motion data, which were subsequently converted into distinct values for motor speed, amplitude, and rhythm for each stimulation trial. 

Bradykinesia assessments were carried out using low- and high-frequency stimulation for the four contact pairs in monopolar settings with the cathode on the contact and the anode on the case [e.g., (Right) 1-C+/(Left) 1-C+]. The labeling of the electrode contacts was standardized among the DBS systems, with contact 1 indicating the most ventral contact level and contact 4 referring to the most dorsal contact level. Threshold amplitudes were determined as 180 Hz for each contact pair and 60 Hz amplitudes were calculated to maintain an equivalent total electrical energy delivered. High-frequency STN-DBS usually ranges from 130 to 185 Hz as part of standard of care and has been demonstrated to acutely worsen gait [13]. Since 180 Hz is in the upper limit of the therapeutic high frequency range, it provided a sharp contrast with 60 Hz for the purpose of the clinical trial that this substudy was conducted under. The Kinesia sensor system provides motor scores in a 0–4 range—similar to the UPDRS- with 0.001 resolution for each movement. It was used to measure 3 components of bradykinesia: speed, amplitude, and rhythm. We focused this analysis on the hand grasp task to reduce the impact that dyskinesia in the ON-medication state could produce within pronation–supination movements. Kinesia sensor scores were normalized by subtracting a given trial’s score by the mean score across all trials for that subject.

The following parameters were adjusted: L-DOPA medication state (ON/OFF), DBS contact (contact 1, 2, 3, or 4), and DBS frequency (60/180 Hz). All DBS contact–frequency combinations were tested first in the OFF-medication state followed by the ON-medication state. The order of the contact–frequency combinations was randomized for each subject prior to the study. The DBS amplitude was adjusted per trial to yield the most optimal clinical effect while limiting side effects. Subjects were blinded to each parameter change and the order of the contact–frequency combinations was randomized. Following each stimulation parameter change, a 10 min rest period was administered to allow for accommodation. To account for the washout period, the OFF-medication state was achieved following an overnight withdrawal of all dopaminergic medications and the OFF-DBS state was achieved following a 50 min wait period. To transition to the ON-medication state, patients were administered 150% of their clinically derived L-DOPA dose (up to 300 mg) using 25 mg/100 mg carbidopa-levodopa tablets followed by a wait period of up to one hour. 

### 2.4. Statistical Analysis

For categorical variables, descriptive statistics (including frequencies and percentages) were implemented. For numerical variables, inferential statistical methods (mean, median) were used and parametric statistical tests (ANOVA) were implemented to determine statistical significance. A linear regression model was implemented to determine the independent impact of medication state (OFF/ON), stimulation frequency (180/60 Hz), and electrode contacts on each sensor-derived bradykinesia value. We converted each categorical variable into a numerical variable for analysis (variable coding). This was followed by an assessment of significant model factors via an ANOVA. This model allowed for the evaluation of the individual impact of each categorical variable on continuous variables (sensor output values). A *p*-value of 0.05 was implemented to determine statistical significance. All statistical analyses were conducted using MATLAB R2020a (Mathworks Inc., Natick, MA, USA). All custom scripts and de-identified; anonymized data are available upon reasonable request to the corresponding author.

## 3. Results

### 3.1. Demographics

We recruited 22 subjects (6 females) for this study. Their mean age was 63.6 ± 9.1 years, and the mean duration of disease was 14.4 ± 7.8 years. Baseline bradykinesia scores in the OFF-medication/OFF-DBS state were measured using the Kinesia sensor. The mean right hand grasping scores were speed: 3.21 ± 0.63; amplitude: 3.62 ± 0.30; and rhythm: 1.64 ± 1.24. The mean left hand grasping scores were speed: 3.04 ± 0.73; amplitude: 3.53 ± 0.42; and rhythm: 1.54 ± 1.10. 

### 3.2. Motor Speed

**Figure 1:**
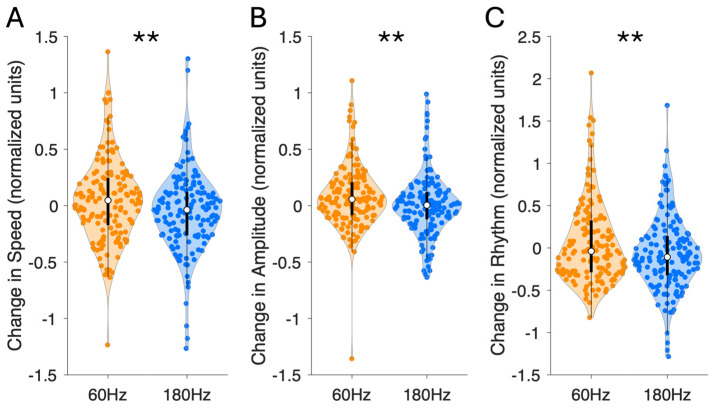
Change in hand movements’ (**A**) speed; (**B**) amplitude; and (**C**) rhythm following 60 Hz (orange) and 180 Hz (blue) subthalamic nucleus deep brain stimulation (*n* = 22 subjects; *n* = 150 60 Hz stimulation trials; *n* = 150 180 Hz stimulation trials). Lower values indicate bradykinesia symptom improvement. One point represents one stimulation trial. Black vertical line represents boxplot, with white circle representing sample median. ** represents significance at *p* < 0.05 level (paired-samples *t*-test).

The mean speed subscore change for 60 Hz stimulation was 0.049 ± 0.030 compared to −0.049 ± 0.029 (please note that lower values indicate symptom improvement, similar to the UPDRS) for 180 Hz (t(299) = 2.340, *p* = 0.020) (Figure 1A). The mixed linear regression model revealed that the medicated state (F = 25.666, *p* < 0.001) and a 180 Hz stimulation frequency (F = 6.636, *p* = 0.011) independently improved motor speed, but not contact pairs (F = 0.271, *p* = 0.846) or stimulation amplitude (F = 1.451, *p* = 0.229) (Table 1). No interactions between contacts and stimulation frequency were found to have a significant relationship with motor speed improvement (Table S1).

### 3.3. Motor Amplitude

The mean amplitude subscore change for 60 Hz stimulation was 0.080 ± 0.022 compared to 0.012 ± 0.022 for 180 Hz (t(299) = 2.102, *p* = 0.036) (Figure 1B). The mixed linear regression model revealed that stimulation amplitude (F = 6.322, *p* = 0.012), a 180 Hz frequency (F = 10.696, *p* = 0.001), and the ON-medication state (F = 9.050, *p* = 0.003) all independently improved motor amplitude, while contact pairs did not (F = 0.038, *p* = 0.990) (Table 1). No interaction effects between contact pairs and stimulation frequency were found (Table S1). 

### 3.4. Motor Rhythm

The mean rhythm subscore change for 60 Hz stimulation was 0.062 ± 0.039 compared to −0.068 ± 0.036 for 180 Hz (t(299) = 2.440, *p* = 0.015) (Figure 1C). The mixed linear regression model revealed that a 180 Hz stimulation frequency (F = 4.363, *p* = 0.038) and ON-medication state (F = 12.527, *p* < 0.001) independently improved rhythmicity, but not contact pairs (F = 0.750, *p* = 0.523) or stimulation amplitude (F = 0.128, *p* = 0.721) (Table 1). We identified significant interaction effects for stimulation applied at contact 2 (B = 1.375, *p* = 0.004) and contact 3 (B = 1.872, *p* < 0.001) (but not contact 4; B = 0.307, *p* = 0.265), which resulted in increased dysrhythmia compared to contact 1. 

## 4. Conclusions

In this study, we implemented data from a STN-DBS trial in an advanced PD cohort to examine the impact of stimulation parameters on upper extremity bradykinesia subcomponents, as measured by wearable sensor technology. We show that 180Hz STN DBS (compared to 60 Hz STN DBS) and dopaminergic medications improved all subcomponents of upper extremity bradykinesia. These results are in line with previous reports showing the effects of DBS and dopaminergics on movement speed and amplitude. The differential impacts of high and low stimulation frequencies warrant further longitudinal investigation as the mean differences between these stimulation modes are small and may be clinically negligible. Low frequency (60 Hz) DBS remains a therapeutic option for patients with gait disorders; however, its response is often variable and not predictable. We previously showed that 60Hz DBS can be utilized early in the programming course of PD patients with gait disorder and that its therapeutic benefit was maintained for long periods [13]. Blumenfeld et al. reported that both 140Hz DBS and 60 Hz DBS improved bradykinesia with an associated reduction in pathological STN beta oscillations [6]. In parallel, it is believed that the targeting of symptom-specific “sweet spots” with STN-DBS results in optimal clinical effects [14,15,16]. This location is usually outside the boundary of the stimulation field generated by most dorsal contacts in accurately placed electrodes [17,18,19,20]. When taken together with our current findings, this promotes some novel programming considerations, including (1) applying 60 Hz stimulation for gait may not hamper bradykinesia response and (2) the avoidance of dorsal contacts may lead to optimal bradykinesia reduction.

Additionally, stimulation improvements likely stem from alterations in neuronal beta oscillatory activity, as local field potential recordings from DBS electrodes revealed a correlation between bradykinesia and the STN beta band oscillatory burst activity of the basal ganglia [21]. Lofredi et al. [22] showed that the time spent in beta bursts of the STN predicted a reduction in movement velocity, thus underscoring the complexity of oscillatory changes in the clinical dimensions of bradykinesia. The reduction in arrhythmicity for both dopaminergics and DBS is not directly linked to sequencing effects, but suggestive of motor timing impairment. This may reflect changes in a different node of the motor system, possibly tied to the cerebellum [3,23]. Recent studies have identified a relationship between DBS and finely tuned gamma oscillations in the STN that may influence bradykinesia improvements [24,25]. Finely tuned gamma activity (40–100 Hz) closely correlates with local neuronal population activity [26]. It is possible that high-frequency STN DBS elicits subharmonic effects that drive the resulting motor effects (e.g., 180 Hz stimulation evoking 90 Hz harmonic activity) [27,28]. 

There are limitations that underlie the findings of this study. First, this trial was conducted on a small, advanced PD cohort, which limits the generalizability of these findings to all subjects with PD. Second, multiple stimulation trials were performed over 2–3 h, and, although stimulation trial parameters were randomized within each subject, we cannot rule out the effects of fatigue. Finally, we cannot exclude that 180Hz stimulation may exhibit a differential response compared to other frequencies used in the standard of care within the high-frequency stimulation range. 

In summary, the use of motion sensors is critical to understanding and measuring treatment responses in PD. Not only do the MDS-UPDRS bradykinesia scores have low reliability, but they are not able to divulge the components of bradykinesia in a systematic manner. As our ability to conduct objective, quantitative assessments on patients grows (especially with the advent of segmented, or directional, DBS leads [29]), engineering DBS studies that incorporate longitudinal sensor assessments while accounting for stimulation dynamics and neurophysiology effects will be important to advance the improvement and application of this treatment clinically. 

## Figures and Tables

**Table 1:** Main effects of ANOVA analysis of the linear regression model, split by each bradykinesia subdomain (speed, amplitude, and rhythm).

	Speed		Amplitude		Rhythm	
	F-Value	*p*-Value	F-Value	*p*-Value	F-Value	*p*-Value
Contact Number	0.272	0.846	0.038	0.990	0.750	0.523
Medication State	25.666	<0.001	9.050	0.003	12.527	<0.001
Stimulation Amplitude	1.451	0.229	6.323	0.013	0.128	0.721
Stimulation Frequency	6.636	0.011	10.696	0.001	4.364	0.038

## Data Availability

Data are contained within the article and Supplementary Materials.

## Supplementary Materials

The following supporting information can be downloaded at: https://www.mdpi.com/article/10.3390/s24134296/s1, Table S1: Main and interaction effects of ANOVA analysis of the linear regression model, split by each bradykinesia subdomain (speed, amplitude, and rhythm).
