# Supplementary material for: Differential Responses to Low- and High-Frequency Subthalamic Nucleus Deep Brain Stimulation on Sensor-Measured Components of Bradykinesia in Parkinson’s Disease
Source: Sensors (Basel). 2024 Jul 2;24(13):4296. doi: 10.3390/s24134296 (PMC11244034; doi:10.3390/s24134296)
Supplement: Supplementary file 1 [file sensors-24-04296-s001.zip › sensors-3025674-supplementary.pdf]

Table S1. Main and interaction effects of ANOVA analysis of the linear regression model, split by each bradykinesia subdomain (speed, amplitude, and rhythm).

	Speed		Amplitude		Rhythm	
	F-value	p-value	F-value	p-value	F-value	p-value
Contact Pairs	0.272	0.846	0.038	0.990	0.750	0.523
Medication State (on)	25.666	**<0.001**	9.050	**0.003**	12.527	**<0.001**
Stimulation Amplitude	1.451	0.229	6.323	**0.013**	0.128	0.721
Stimulation Frequency (180Hz)	6.636	**0.011**	10.696	**0.001**	4.364	**0.038**
Contact Pairs : MedState	1.934	0.124	0.837	0.475	1.885	0.132
Contact Pairs: StimAmp	0.711	0.546	0.339	0.797	3.100	**0.027**
MedState : StimAmp	1.506	0.221	1.055	0.305	3.015	0.084
Contact Pairs: StimFreq	1.252	0.291	0.594	0.620	4.296	**0.006**
MedState: StimFreq	2.058	0.153	0.792	0.374	1.628	0.203
StimAmp : StimFreq	1.077	0.300	0.961	0.328	0.003	0.958
Contact Pairs : MedState : StimAmp	0.066	0.978	0.951	0.416	0.614	0.607
Contact Pairs : MedState : StimFreq	0.159	0.924	0.991	0.398	0.302	0.824
Contact Pairs : StimAmp : StimFreq	0.752	0.522	0.599	0.616	0.828	0.479
MedState : StimAmp : StimFreq	0.388	0.534	0.303	0.582	0.283	0.595
Contact Pairs : MedState : StimAmp : StimFreq	0.301	0.825	1.143	0.332	0.500	0.683
